# Weaning Alters Intestinal Gene Expression Involved in Nutrient Metabolism by Shaping Gut Microbiota in Pigs

**DOI:** 10.3389/fmicb.2020.00694

**Published:** 2020-04-17

**Authors:** Qingwei Meng, Zhang Luo, Chunyu Cao, Shishuai Sun, Qingquan Ma, Zhongyu Li, Baoming Shi, Anshan Shan

**Affiliations:** Institute of Animal Nutrition, Northeast Agricultural University, Harbin, China

**Keywords:** weaning transition, transcriptome, gut microbiota, short-chain fatty acids, pigs

## Abstract

Weaning transition usually impairs intestinal architecture and functions and results in gut-associated disorders in pigs. Understanding the changes in intestinal transcriptome and gut microbiota during weaning transition is important for elucidating the underlying mechanism of weaning stress. In the present study, we performed RNA-seq to determine the changes in intestinal transcriptome and 16S rRNA sequencing to measure the gut microbiota changes in the weaning transition. Transcriptome results indicated that weaning transition altered intestinal gene expression involved in nutrient transport and metabolism. Regarding fatty metabolism, fatty acid-binding protein 1 (*FABP1*), acyl-CoA dehydrogenase (*ACADSB*), and carnitine palmitoyltransferase 2 (*CPT2*) expression in the intestine was decreased by weaning. Genes related to bile acid metabolism were increased by weaning, including *FABP6*, farnesoid X receptor (*FXR* or *NR1H4*) and organic solute transporter-α (*SLC51A*). In addition, genes associated with oxidative stress were altered by weaning transition, including decreased catalase (*CAT*) and lactate dehydrogenase (*LDHA*) and increased glutathione peroxidase 2 (*GPX2*) and superoxide dismutase 3 (*SOD3*). Results of microbiota composition showed that the Firmicutes abundance and Firmicutes/Bacteroidetes ratio were increased and that the Proteobacteria abundance in the fecal microbiota was decreased by the weaning process; during the weaning transition, the *Bacteroides* and *Fusobacterium* abundances decreased markedly, and these bacteria nearly disappeared, while the *Prevotella* abundance showed a marked increase. Moreover, the levels of the microbial metabolites butyrate and acetate increased with changes in gut microbiota composition. In addition, predictive metagenome by PICRUSt analysis showed that the pathways related to D-glutamine and D-glutamate metabolism, citrate cycle (TCA cycle), peroxisome proliferators-activated receptor (PPAR) signaling, alpha-linolenic acid metabolism were decreased and the pathway related to retinol metabolism was increased in the gut microbiota of piglets during weaning transition. Our results showed that early weaning alters intestinal gene expression involved in nutrient metabolism, which may be due to the changes in microbiota composition.

## Introduction

The weaning transition in pigs involves a sudden dietary shift from maternal milk to completely solid food, which unlike in humans (Smith et al., 2010; Merrifield et al., 2013). In addition, under commercial conditions, early weaning involves abrupt separation from sows, resulting in a sudden move to a new environment at a much younger age than would occur in natural conditions (Hu et al., 2013; Pluske, 2016). Many studies have demonstrated that early weaning impairs the gastrointestinal architecture and function of pigs, resulting in gut-associated disorders, including diarrhea (Weary et al., 2008), increased intestinal permeability (Wijtten et al., 2011; Hu et al., 2013), inflammation (Pié et al., 2004), and oxidative stress (Yin et al., 2014). Furthermore, weaning transition activates stress and inflammation signaling pathways and results in abnormal expression of intestinal genes and proteins in pigs (Moeser et al., 2007; Wang et al., 2008; Hu et al., 2013). However, the exact underlying mechanisms associated with the effects of weaning transition on gut function remain unclear. The transcriptome is the complete set of transcripts in a cell, and their quantity, for a specific developmental stage or physiological condition (Wang et al., 2009). RNA-seq is a high-throughput approach to transcriptome profiling that uses next-generation sequencing technologies for the analysis of gene expression (Wang et al., 2009; Xia et al., 2017). RNA-seq eliminates several problems associated with microarray technologies, including its restriction to known genes and limited dynamic range of detection (Ozsolak and Milos, 2011; Xia et al., 2017). However, there is a lack of characterization on RNA-seq analysis in the intestine of piglets under the weaning condition.

An increasing number of human and animal studies have demonstrated that the intestinal microbiota is closely associated with various diseases, such as inflammatory bowel disease, cancer, obesity, and insulin resistance (Round and Mazmanian, 2009; Kanauchi et al., 2013; Liu et al., 2017). In recent years, many studies indicated that intestinal microbiota is associated with gut development, diarrhea (Yang et al., 2017b), fat deposition (Jiang et al., 2016; Yang et al., 2018), feed efficiency, and growth performance in pigs (Han et al., 2017; Yang et al., 2017a; Quan et al., 2019). Gut microbiota benefits the host in different ways, such as digestion and fermentation of carbohydrates, production of short-chain fatty acids (SCFAs), maintenance of normal functions of the intestinal villi, regulation of the immune responses, and protection from pathogenic bacteria (Gresse et al., 2017; Levy et al., 2017). In newborn mammals, microbes colonize all body surfaces at birth and participate in the development of the immune system and metabolic process (Bian et al., 2016). Shortly after birth, the colonizing intestinal microbiota is first shaped by the dietary and immunological components of milk and follows a developmental process that is characterized by the presence of age-specific bacterial species (Le Doare et al., 2018; Al Nabhani et al., 2019). During weaning, the introduction of solid food leads to a new phase in the development of the microbiota, characterized by a large increase in bacterial numbers, and evolution toward a composition that is associated with adult individuals (Frese et al., 2015). Among the physiological and intestinal factors impacted by the weaning transition, gut microbiota disruption is likely to be recognized as one of the keys leading to weaning stress. Moreover, gut microbial metabolite has been shown to play multiple roles in intestinal development, immunity function, and regulation of host digestion and metabolism (Levy et al., 2017; Federici, 2019). SCFAs, primarily acetate, propionate, and butyrate, are organic acids produced by microbial fermentation of mainly undigested dietary carbohydrates (Hamer et al., 2008). SCFAs are important energy sources for intestinal epithelial cells and have been demonstrated to maintain intestinal homeostasis and afford protection against intestinal inflammation and oxidative stress (Wu et al., 2017). Dietary factors play an important role in the shaping of microbial populations and SCFAs production. During the weaning process, a dietary shift from solely sow milk to a complete feed-based diet may shape the intestinal microbiota composition and alter SCFAs production, which may regulate intestinal gene expression. In the present study, suckling piglets and piglets at 7 days postweaning were used to construct a weaning model to investigate the changes of the intestinal transcriptome, gut microbiota and SCFAs composition during weaning transition. The postweaning period is a critical period in the swine industry because weaning stress leads to diarrhea, growth inhibition and even death (Lin et al., 2019). The present study will provide a theoretical basis for the development of effective nutritional strategies and feeding approaches for the alleviation of weaning stress.

## Materials and Methods

### Ethics Statement

Animal care and treatment were complied with the standards described in the guidelines for the care and use of laboratory animals of the Northeast Agricultural University [NEAU-(2011)-9]. All the animal experimental procedures were approved by the Ethical and Animal Welfare Committee of Heilongjiang Province, China.

### Animal Management and Sample Collection

**FIGURE 1:**
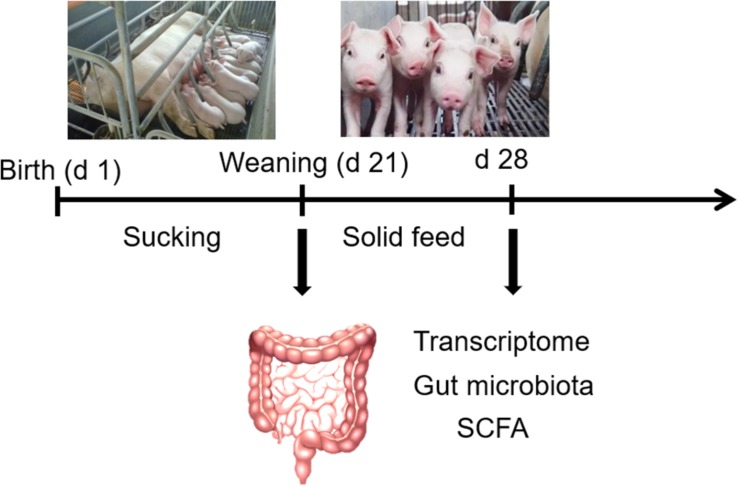
The schematic diagram of experimental design.

Six litters (Yorkshire × Duroc) with 9–11 piglets were selected. The six sows (parity 3–4) were fed the same diets during gestation and lactation and the piglets were weaned at 21 days of lactation. On the morning of the day of weaning (21 days of age), one male sucking piglet approaching average weight was selected from each litter, and a total of six sucking piglets (average BW 5.35 ± 0.24 kg) were slaughtered for sample collection. Then, piglets from each litter were earmarked and transferred to the nursing room. All piglets were allowed free access to water and fed the same creep feed. At 7 days postweaning (28 days of age), one male weaned piglet was selected from each litter for slaughter. The piglets were slaughtered after general anesthesia by intra-arterial injection of 200 mg/kg pentobarbital sodium (Sigma-Aldrich, St Louis, MO, United States). Fresh feces (rectal contents) were collected and stored immediately at −80°C for microbiota and SCFAs analysis. Jejunum tissue was rinsed and stored immediately at −80°C for further analysis. The schematic diagram of the experimental design is shown in Figure 1.

### RNA-Seq Analysis

Total RNA was isolated from intestinal tissue using Trizol reagent (Invitrogen, United States). The quantity and purity of total RNA were analyzed with an Agilent Technologies 2100 bioanalyzer (Agilent, United States). The cDNA library was constructed and then sequenced with an Illumina 4000 sequence platform, producing 150-bp paired-end reads. A total of six samples with three biological replicates in each treatment were conducted to RNA-seq analysis. The RNA-seq data of these six samples have been deposited in the Gene Expression Omnibus (GEO) database under accession numbers of GSM2868043, GSM2868044, GSM2868045, GSM2868049, GSM2868050, and GSM2868051. Sample reads were aligned to the Ensembl pig reference genome using TopHat. StringTie and Ballgown were used to estimate the gene expression levels of each sample. The gene expression levels were estimated by calculating the value of fragments per kilobase of exon per million reads mapped (FPKM). The differentially expressed genes (DEGs) were judged with log2 (fold-change) >1 or log2 (fold-change) <−1 and *P-*value <0.05. STRING database was used to analyze the interaction networks between proteins encoded by DEGs. Gene ontology (GO) enrichment analysis and KEGG pathway analysis were performed by using the GOseq R package and KOBAS software, respectively.

### qRT-PCR Validation of RNA-Seq Analysis

The total RNA (2 μg) from each sample was converted into cDNA for RT-PCR using the Prime Script RT reagent Kit (TaKaRa Bio Catalog, Beijing, China). The RT-PCR was performed using the SYBR Green I Kit (TaKaRa Bio Catalog, Beijing, China). For analyses on an ABI PRISM 7500 SDS thermal cycler, PCR reactions were performed with 2.0 μL of first-strand cDNA and 0.4 μL of sense and anti-sense primers in a final volume of 20 μL. Samples were centrifuged briefly and run on the PCR machine using the default fast program (1 cycle at 95°C for 30 s, 40 cycles of 95°C for 5 s and 60 °C for 34 s). All the PCR reactions were performed in triplicate. The primer sequences are shown in Supplementary Table 1. The relative gene expression levels were calculated using the 2^–ΔΔCt^ method normalizing to glyceraldehyde-3-phosphate dehydrogenase (*GAPDH*) expression (Livak and Schmittgen, 2001).

### 16S rRNA Sequencing

DNA from fecal samples was isolated using the Stool DNA Kit (Omega, United States) and then eluted in 50 μL of elution buffer by a modification of the procedure. The primers (F: 5′-ACTCCTACGGGAGGCAGCAG-3′; R: 5′-GGACTACHVGGGTWT-CTAAT-3′) were used in the PCR amplification for the V3–V4 region of the bacterial 16S rRNA gene. PCR analysis was performed in triplicate with 25-μL reactions containing 12.5 μL of PCR premix, 2.5 μL of each primer, 25 ng of template DNA, and PCR-grade water to equalize the final volumes. The PCR products were confirmed with 2% agarose gel electrophoresis, purified with AMPure XT beads (Beckman Coulter Genomics, Danvers, MA, United States) and then quantified by Qubit (Invitrogen, CA, United States). The amplicon pools were prepared for sequencing, and the size and quantity of the amplicon library were assessed on an Agilent 2100 bioanalyzer (Agilent, United States) and with the Library Quantification Kit for Illumina (Kapa Biosciences, Woburn, MA, United States).

Amplicon libraries were sequenced on an Illumina MiSeq platform according to the manufacturer’s recommendations, which provided by LC-Bio. The raw paired-end reads each sample were truncated by removing the barcode and primer sequence. Quality filtering on the raw tags was performed to obtain high-quality clean tags using FASTQC software (version 0.10.0). The high-quality clean sequences were assigned to OTUs at 97% similarity. Representative sequences were selected for each OUT and taxonomic data were assigned to each representative sequence using the RDP classifier. Principal coordinate analysis (PCoA) plots were generated according to the unweighted UniFrac distance metrics. The number of observed species and the indices of Chao 1 (species richness), Shannon and Simpson (diversity) were calculated to estimate alpha diversity. Microbial functions were examined by predicting the metagenomes using PICRUSt (Phylogenetic investigation of communities by reconstruction of unobserved states) analysis based on high-quality sequences (Langille et al., 2013).

### SCFAs Analysis

SCFAs levels were measured by gas chromatography (Shimadzu GC-2010, Japan). Samples analysis was performed by mixing 2 g of the feces with 2 mL of ultrapure water. Then, the samples were centrifuged at 10,000 rpm for 10 min under 4°C. The supernatant was passed through a 0.22-μm filter, and this procedure was repeated three times. The final supernatant was supplemented at a ratio of 5:1 with 25% (w/v) metaphosphoric acid for the SCFAs assay (Zhang et al., 2017).

### Statistical Analysis

The data of SCFAs levels and qRT-PCR validation were analyzed by using SPSS 18.0 (IBM-SPSS Inc., Chicago, IL, United States). The significance of the difference between two groups was analyzed by Student’s *t*-test. Differences were considered significant if *P*-value <0.05, and *P*-value between 0.05 and 0.10 were considered a trend. The results are presented as the mean values and the standard error of the mean (SEM).

## Results

### Summary of RNA-Seq Analysis

The main characteristics of the six libraries (three biological replicates in each treatment) are shown in Supplementary Table 2 and contained 49,418,207 raw reads on average. After removing adaptor, low-quality and ambiguous sequences, an average of 45,625,929 valid clean reads remained. Across the six samples, 98.54% of the raw data were valid data and 77.89% of the valid reads mapped to the database, including 49.83% unique mapped reads and 28.06% multi-mapped reads, as shown in Supplementary Table 3.

### Differential Gene Expression

**FIGURE 2:**
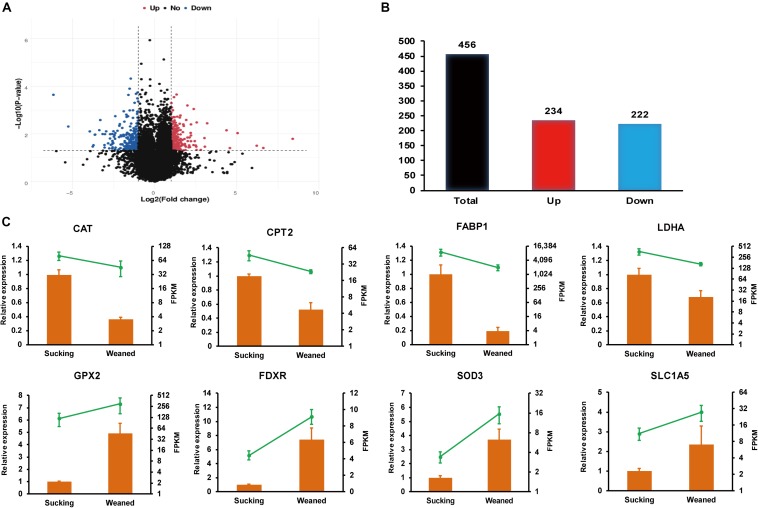
RNA-seq analysis of the intestines of sucking piglets and weaned piglets. **(A)** Volcano plot of DEGs; fold-changes were calculated as weaned piglets/sucking piglets. **(B)** Number of up-regulated and down-regulated DEGs. **(C)** Relative expression levels from qRT-PCR and FPKM from RNA-seq analysis; the mRNA levels of the selected genes were analyzed by qRT-PCR and normalized to those of *GAPDH*; all values are expressed as the means ± SEMs (*n* = 3).

In the present study, *P*-value <0.05 and | log2 (fold-change)| > 1 were used as standards to identify DEGs. Volcano plots (Figure 2A) were used to visualize the distribution of DEGs between sucking piglets and weaned piglets. In the volcano plot, the blue dots represent down-regulated genes, and red dots represent up-regulated genes. There are 456 DEGs between sucking piglets and weaned piglets, including 234 up-regulated genes and 222 down-regulated genes (weaned/sucking, Figure 2B and Supplementary Table 4).

### qRT-PCR Validation

Relative expression levels from qRT-PCR and FPKM values from the transcriptomic data are shown in Figure 2C. Eight genes involved in nutrient metabolism and oxidative stress were selected, and the RNA-seq data were further validated by the qRT-PCR assay. The relative expression levels of catalase (*CAT*), carnitine palmitoyltransferase 2 (*CPT2*), fatty acid-binding protein 1 (*FABP1*) and lactate dehydrogenase (*LDHA*) were significantly decreased in weaned piglets compared to sucking piglets (*P* < 0.05). In addition, the relative expression levels of glutathione peroxidase 2 (*GPX2*), ferredoxin reductase (*FDXR*) and superoxide dismutase 3 (*SOD3*) were significantly increased in weaned piglets compared to sucking piglets (*P* < 0.05). The relative expression level of solute carrier family 1, member 5 (*SLC1A5*) was not significantly affected by the weaning process but showed an increasing trend (*P* = 0.08). Overall, the expression trend of most genes validated by qRT-PCR was in accordance with the results from RNA-seq analysis. The differences in the magnitude of fold-change were due to differences in the detection sensitivity of the two methods (Wang et al., 2009).

### GO Enrichment Analysis of DEGs

**FIGURE 3:**
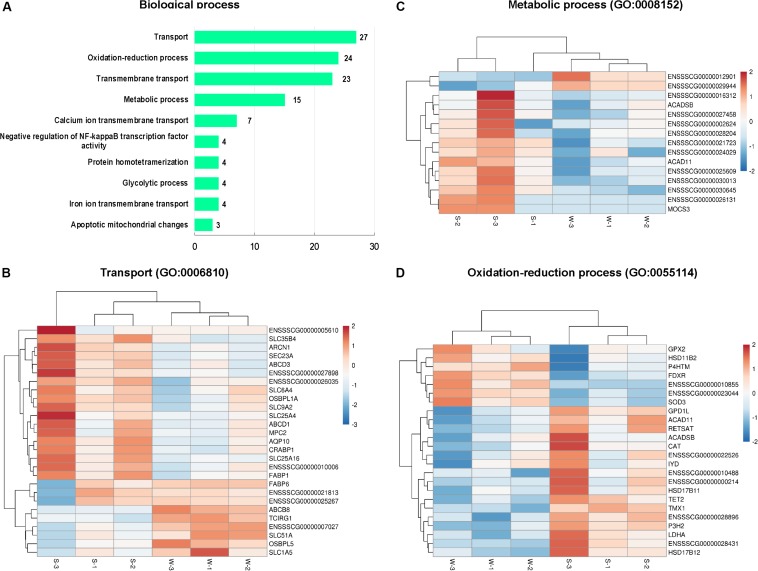
Classification of biological process in the GO functional enrichment analysis of DEGs. **(A)** The top 10 terms in the biological process category. **(B)** Hierarchical clustering heat maps of DEGs in the GO term of transport (GO: 0006810). **(C)** Hierarchical clustering heat maps of DEGs in the GO term of metabolic process (GO: 0008152). **(D)** Hierarchical clustering heat maps of DEGs in the GO term of the oxidation-reduction process (GO: 0055114). S, sucking piglets; W, weaned piglets.

In the present study, GO enrichment analysis was performed with 456 DEGs between sucking piglets and weaned piglets. A total of 251 GO terms were enriched, including 145 biological process terms, 17 cellular component terms and 89 molecular function terms (Supplementary Table 5). The top 10 GO terms belong to the category of the biological process are shown in Figure 3A (based on the number of genes). GO terms associated with transport, oxidation-reduction process, and transmembrane transport were predominant, followed by metabolic process, negative regulation of NF-kappaB transcription factor activity, glycolytic process and apoptotic mitochondrial changes. In the GO term of the transport (GO: 0006810), 27 genes were enriched, as shown in Figure 3B, such as fatty acid-binding protein (*FABP1* and *FABP6*), cellular retinoic acid binding protein 1 (*CRABP1*), mitochondrial pyruvate carrier 2 (*MPC2*), solute carrier family (*SLC1A5*, *SLC25A16*, *SLC25A4*, *SLC35B4*, *SLC51A*, *SLC6A4*, and *SLC9A2*). In the GO term of the metabolic process (GO: 0008152), 15 genes were enriched (Figure 3C), including acyl-CoA dehydrogenase family member 11 (*ACAD11*), acyl-CoA dehydrogenase (*ACADSB*), molybdenum cofactor synthesis 3 (*MOCS3*). In the GO term of the oxidation-reduction process (GO: 0055114), 24 genes were enriched (Figure 3D), including antioxidant enzymes (*CAT*, *GPX2*, *SOD3*), *FDXR*, hydroxysteroid dehydrogenase family (*HSD11B2*, *HSD17B11*, *HSD17B12*), and *LDHA*. The top 10 GO terms in the cellular component category are shown in Supplementary Figure 1A, including membrane, peroxisome, lipid particle, and nucleosome. A total of 146 genes were enriched in the GO term of the membrane (GO: 0016020), as shown in Supplementary Figure 1B.

**FIGURE 4:**
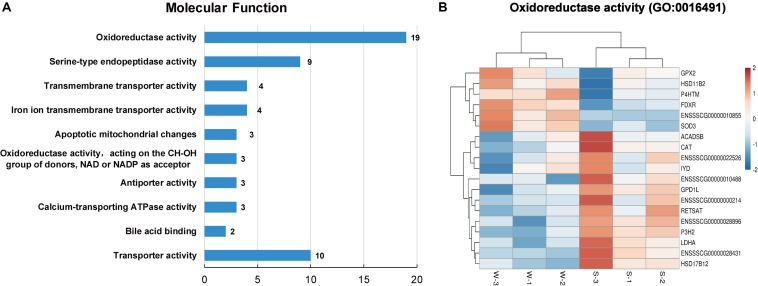
Classification of molecular function in the GO functional enrichment analysis of DEGs. **(A)** The top 10 terms in the molecular function category. **(B)** Hierarchical clustering heat maps of DEGs in the GO term of oxidoreductase activity (GO: 0016491). S, sucking piglets; W, weaned piglets.

**FIGURE 5:**
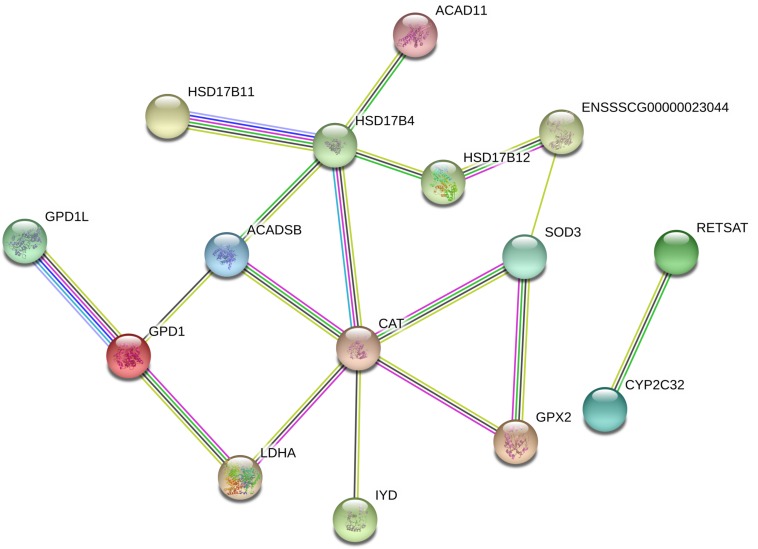
Interaction networks of proteins encoded by DEGs related to oxidative stress. Line colors represent different evidentiary sources for each interaction: light blue, from curated databases; purple, experimental; green, gene neighborhood; red, fusion; blue, gene cooccurrence; yellow, text mining; black, coexpression; light purple, protein homology. Disconnected nodes are not shown in the network.

As shown in Figure 4A, the top 10 GO terms belong to molecular functions, including oxidoreductase activity, transporter activity, and bile acid binding. There are 19 genes in the GO term of oxidoreductase activity, as shown in Figure 4B. These genes are similar to the genes that were enriched in the GO term of oxidation-reduction process (GO: 0055114), belonging to biological process. In these two GO terms related to oxidative stress, a total of 24 genes were enriched, with 7 up-regulated genes, namely, *GPX2*, hydroxysteroid 17-beta dehydrogenase 12 (*HSD11B2*), prolyl 4-hydroxylase (*P4HTM*), *FDXR*, *ENSSSCG00000010855*, *ENSSSCG00000023044* and *SOD3*, and 17 down-regulated genes, including *ACAD11*, *ACADSB*, *CAT*, iodotyrosine deiodinase (*IYD*), tet methylcytosine dioxygenase 2 (*TET2*), and *LDHA* (Figures 3D, 4B). In addition, the interaction networks of proteins encoded by these genes were predicted and constructed using the STRING database. Fifteen proteins were found to interact with other proteins. As shown in Figure 5, CAT interacted with six proteins: ACADSB, GPX2, 17β-hydroxysteroid dehydrogenase type 4 (HSD17B4), IYD, LDHA, and SOD3; glycerol-3-phosphate dehydrogenase 1 (GPD1) interacted with three proteins: ACADSB, glycerol-3-phosphate dehydrogenase 1 like (GPD1L), and LDHA; ACADSB interacted with three proteins: CAT, GPD1, and HSD17B4; GPX2 interacted with CAT and SOD3.

**FIGURE 6:**
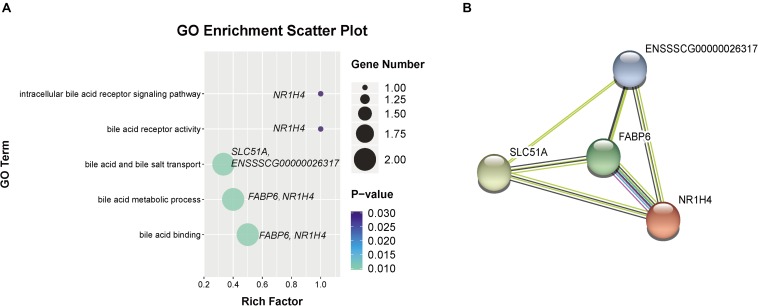
Go terms related to bile acid metabolism. **(A)** GO terms related to bile acid metabolism were increased by weaning. **(B)** Interaction networks of proteins encoded by DEGs related to bile acid metabolism. Line colors represent different evidentiary sources for each interaction: light blue, from curated databases; purple, experimental; green, gene neighborhood; red, fusion; blue, gene cooccurrence; yellow, text mining; black, coexpression; light purple, protein homology. Disconnected nodes are not shown in the network.

In addition, we observed five GO terms related to bile acid metabolism (as shown in Figure 6A), including bile acid metabolic process (GO:0008206), bile acid and bile salt transport (GO:0015721), intracellular bile acid receptor signaling pathway (GO:0038185), bile acid binding (GO:0032052) and bile acid receptor activity (GO:0038181). There are four genes were enriched in these GO terms, including *FABP6*, farnesoid X receptor (*FXR* or *NR1H4*), *SLC51A*, and *ENSSSCG00000026317*, which were increased in weaned piglets. Furthermore, we found the proteins encoded by these genes were markedly interacted with each other by STRING analysis (Figure 6B).

### KEGG Pathway Analysis of DEGs

In the KEGG pathway analysis, 14 pathways were identified (Table 1). There were two pathways related to nutrient absorption, including mineral absorption and the sulfur relay system, and six pathways related to nutrient metabolism, including alanine, aspartate, and glutamate metabolism; arginine and proline metabolism; glyoxylate and dicarboxylate metabolism; fatty acid metabolism; the peroxisome proliferators-activated receptor (PPAR) signaling pathway; and nitrogen metabolism. In addition, five pathways related to nutrient synthesis and digestive enzyme secretion were enriched, including pancreatic secretion, salivary secretion, biosynthesis of amino acids, and folate biosynthesis.

**TABLE 1:** KEGG pathway enrichment of DEGs.

Pathway ID	Pathway name	Down-regulated genes	Up-regulated genes	*P*-value
ko04978	Mineral absorption	ENSSSCG00000016040, ENSSSCG00000027157, ENSSSCG00000027221, ENSSSCG00000029221, S100G, TRPV6	ENSSSCG00000025267	0.0001
ko04122	Sulfur relay system	MOCS2, MOCS3	ENSSSCG00000001596	0.0021
ko00250	Alanine, aspartate and glutamate metabolism	ABAT, CPS1, ENSSSCG00000002797, GLUL	GLS	0.0025
ko04972	Pancreatic secretion	BST1, CELA2A, ENSSSCG00000027898,	ENSSSCG00000025267, ITPR3, PLA2G2D	0.0173
ko00330	Arginine and proline metabolism	CPS1, ENSSSCG00000002797, GLUL	ENSSSCG00000010855, GLS	0.0192
ko01230	Biosynthesis of amino acids	CPS1, ENSSSCG00000002797, GLUL, PGK1	ENSSSCG00000010855	0.0210
ko00630	Glyoxylate and dicarboxylate metabolism	CAT, GLUL, HYI	–	0.0231
ko01212	Fatty acid metabolism	ACADSB, CPT2, ENSSSCG00000030645	ENSSSCG00000029944	0.0240
ko03320	PPAR signaling pathway	CPT2, ENSSSCG00000030645, FABP1	FABP6, OLR1	0.0268
ko04970	Salivary secretion	BST1, ENSSSCG00000027898, KCNN4, TRPV6	ITPR3	0.0268
ko04727	GABAergic synapse	ABAT, ENSSSCG00000000808, GLUL,	GLS, GNG11	0.0268
ko00790	Folate biosynthesis	MOCS2	ENSSSCG00000001596	0.0371
ko00910	Nitrogen metabolism	CPS1, GLUL		0.0371
ko04726	Serotonergic synapse	CASP3, ENSSSCG00000010488, SLC6A4	GNG11, ITPR3	0.0443

*DEGs, differentially expressed genes between the control treatment and resveratrol treatment.*

### Summary of Microbiota Analysis (16S rRNA)

From all the samples (5–6 biological replicates in each treatment), 397,856 quality sequences were obtained after quality filtering. On average, 36,169 sequences in each sample with reading lengths than 300 bp were obtained, as shown in Supplementary Table 6.

### Differences in Microbiota Composition

**FIGURE 7:**
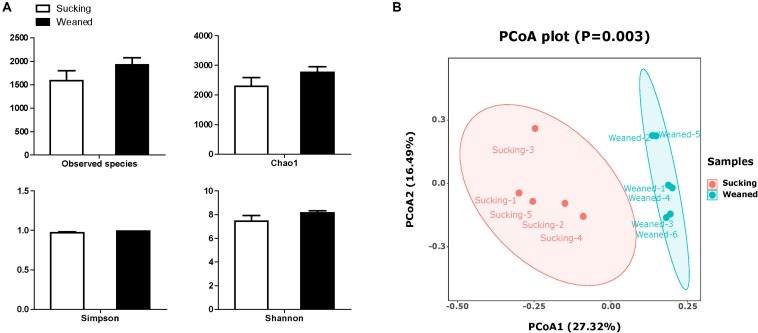
Alpha diversity and beta diversity of the gut microbiota of sucking piglets and weaned piglets. **(A)** The number of observed species and Shannon, Simpson and Chao1 indices of the gut microbiota. All values are expressed as the means ± SEMs (*n* = 5–6). **(B)** Principal coordinates analysis (PCoA, Bray–Curtis distance) plot of the gut microbiota.

**FIGURE 8:**
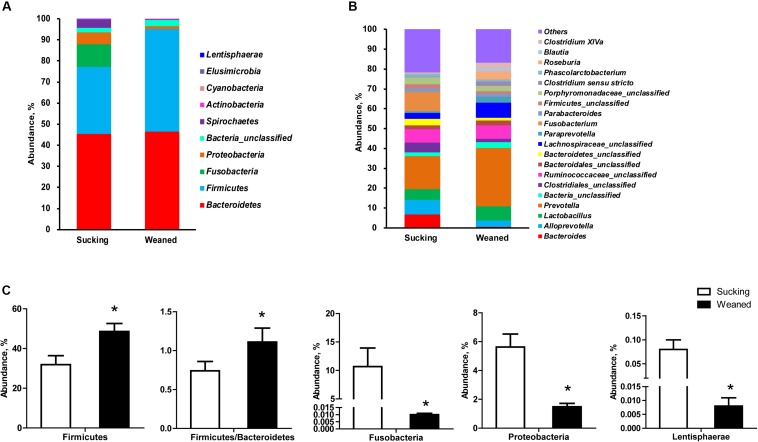
Gut microbiota composition of sucking piglets and weaned piglets. **(A)** Gut microbiota composition at the phylum level. **(B)** Gut microbiota composition at the genus level. **(C)** Different microbes at the phylum level between sucking piglets and weaned piglets. All values are expressed as the means ± SEMs (*n* = 5–6). *P < 0.05.

The number of observed species and indices of Shannon, Simpson, and Chao1 did not differ between sucking and weaned piglets (Figure 7A). The results of unweighted Unifrac distance-based PCoA showed that the microbiota compositions of sucking piglets and weaned piglets were independently distributed (Figure 7B). At the phylum level (Figure 8A), the fecal microbiota of sucking piglets was composed of Bacteroidetes (45.37 ± 4.76%), Firmicutes (31.93 ± 4.51%), Fusobacteria (10.69 ± 3.35%), Proteobacteria (5.63 ± 0.90%), Spirochaetes (3.79 ± 0.95%) and Actinobacteria (0.34 ± 0.10%). In comparison, the fecal microbiota of weaned piglets was composed of Bacteroidetes (46.51 ± 3.80%), Firmicutes (48.56 ± 4.04%), Proteobacteria (1.48 ± 0.24%), Spirochaetes (0.17 ± 0.04%) and Actinobacteria (0.27 ± 0.04%). The abundance of Firmicutes and the Firmicutes/Bacteroidetes ratio were increased (*P* < 0.05) by the weaning transition (Figure 8C). The population sizes of Fusobacteria, Proteobacteria and Lentisphaerae were decreased (*P* < 0.05) by the weaning transition. Notably, the percentage of Fusobacteria showed a marked decrease from 10.69 ± 3.35% to 0.01 ± 0.001%; these bacteria nearly disappeared after weaning.

At the genus level (Figure 8B), *Fusobacterium* and *Prevotella* were the most prevalent genera in sucking piglets, followed by *Alloprevotella*, Ruminococcaceae-unclassified, *Bacteroides* and *Lactobacillus.* In weaned piglets, *Prevotella*, Lachnospiraceae*-*unclassified, and *Lactobacillus* were the most prevalent genera in weaned piglets, followed by Ruminococcaceae-unclassified, *Roseburia, Alloprevotella* and *Paraprevotella*.

**FIGURE 9:**
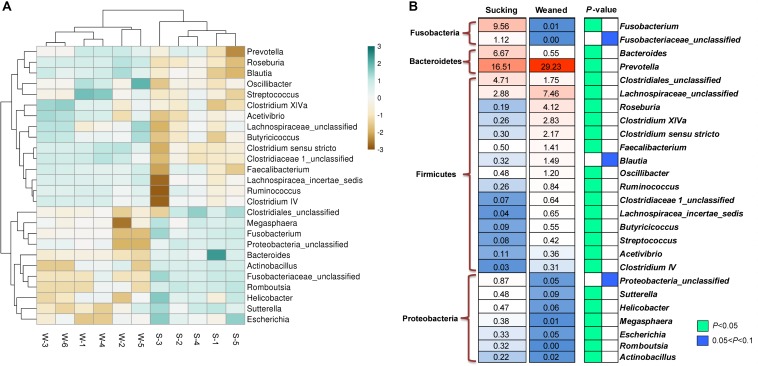
Different microbes at the genus level between sucking piglets and weaned piglets. **(A)** Hierarchical clustering heat maps of different genera between sucking piglets and weaned piglets; S, sucking piglets; W, weaned piglets. **(B)** Statistical analysis of different genera between sucking piglets and weaned piglets. All values are expressed as the means ± SEMs (*n* = 5–6). *P < 0.05.

As shown in Figures 9A,B, the abundances of the genera *Prevotella*, Lachnospiraceae-unclassified, *Roseburia*, *Clostridium* XlVa, *Streptococcus*, *Faecalibacterium*, *Oscillibacter*, *Ruminococcus*, etc. were increased (*P* < 0.05) by weaning transition. The abundance of *Prevotella* showed a significant increase from 16.51 ± 4.81% in sucking piglets to 29.23 ± 3.62% in weaned piglets. In comparison, the abundances of the genera *Bacteroides*, *Fusobacterium*, Clostridiales-unclassified, *Sutterella*, *Helicobacter*, *Megasphaera*, *Escherichia*, *Romboutsia*, and *Actinobacillus* were decreased by the weaning process (*P* < 0.05). The proportion of *Bacteroides* decreased from 6.67 ± 1.06% in sucking piglets to 0.55 ± 0.1% in weaned piglets. In addition, the proportion of *Fusobacterium* decreased from 9.56 ± 2.59% in sucking piglets to 0.01 ± 0.003% in weaned piglets.

### Predictive Metagenome Profiling

**FIGURE 10:**
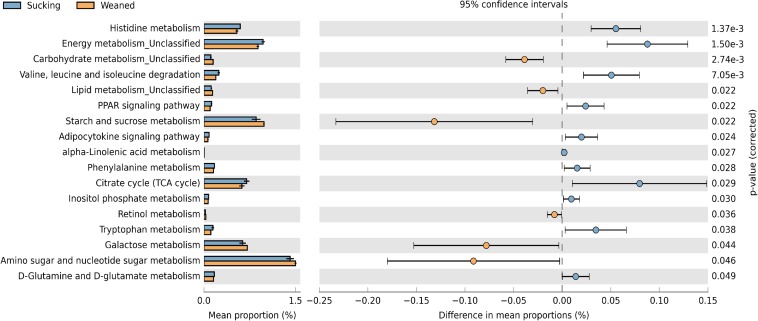
Predictive functional profiles generated from 16S rRNA marker gene sequences using PICRUSt. Functional profiles were generated based on KEGG ortholog prediction and collapsed into higher pathways (L3), according to the KEGG pathway database.

The changes in the presumptive functions of the gut microbiota of piglets during weaning were examined by predicting the metagenomes using PICRUSt as shown in Figure 10. The pathways were involved in the metabolism of amino acid, carbohydrate, and lipid. Regarding amino acid metabolism, pathways related to histidine metabolism, valine, leucine and isoleucine degradation, phenylalanine metabolism, tryptophan metabolism, D-glutamine, and D-glutamate metabolism were decreased (*P* < 0.05) in weaned piglets. Regarding carbohydrate metabolism, pathways related to starch and sucrose metabolism, galactose metabolism, amino sugar and nucleotide sugar metabolism were increased (*P* < 0.05) and pathways related to citrate cycle (TCA cycle), inositol phosphate metabolism were decreased (*P* < 0.05) in weaned piglets. Regarding lipid metabolism, pathways related to alpha-linolenic acid metabolism, PPAR signaling, adipocytokine signaling were decreased (*P* < 0.05) in weaned piglets. In addition, we observed that the pathway related to retinol metabolism was increased (*P* < 0.05) in weaned piglets.

### SCFAs Concentrations in the Feces

**FIGURE 11:**
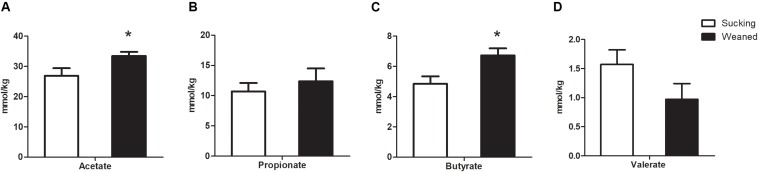
Short-chain fatty acids (SCFAs) concentrations in the feces of sucking piglets and weaned piglets. **(A)** Acetate. **(B)** Propionate. **(C)** Butyrate. **(D)** Valerate. All values are expressed as the means ± SEMs (*n* = 5–6). *P < 0.05.

In addition, the SCFAs concentrations in the feces samples (*n* = 5–6 in each treatment) of piglets were measured. The concentrations of acetate and butyrate were higher (*P* < 0.05) in the feces of weaned piglets than that in sucking piglets (Figure 11). The weaning process failed (*P* > 0.05) to influence the concentrations of propionate and valerate in the feces.

## Discussion

To further elucidate the underlying molecular mechanism responsible for the weaning process, we compare intestinal transcriptome, gut microbiota and SCFAs levels of sucking piglets and weaned piglets. In the present study, a total of 456 DEGs were found between sucking piglets and weaned piglets, including 234 up-regulated genes and 222 down-regulated genes. To validate the DEGs identified by RNA-seq analysis, we measured the expression levels of eight genes by qRT-PCR. A comparison of the results obtained using the two methods revealed similar trends, confirming the validity of the methods. According to the GO analysis, we found that most of the DEGs were related to nutritional transportation, transmembrane transportation and metabolic processes. The KEGG pathway enrichment analysis showed that fatty acid metabolism and PPAR signaling pathways were significantly influenced by the weaning process. In these pathways, *ACADSB, CPT2*, and *FABP1* were down-regulated in weaned piglets. *ACADSB* is a member of the acyl-CoA dehydrogenase family of enzymes that catalyze the dehydrogenation of acyl-CoA derivatives in the metabolism of fatty acids (Jiang et al., 2018). *FABP1* plays a crucial role in intracellular fatty acid transport by binding and properly targeting long-chain fatty acids to the correct metabolic sites (Samulin et al., 2008). *CPT2* is a mitochondrial membrane protein that is responsible for transporting long-chain fatty acids to the mitochondrial inner membrane for fatty acid β-oxidation (Meng et al., 2018). These down-regulated genes indicated that fatty acid transport and oxidation in the gut of piglets may be inhibited by the weaning process. Sow milk is highly palatable and digestible, whereas feed is rough, solid, less tasty, and not as easily digested (Lalles et al., 2004). The introduction of solid food serves as the major challenge in the weaning process of piglets, causing both physical and metabolic reconstructions in the intestine (Wang et al., 2019b), which may induce the changes in intestinal gene expression. On the other hand, previous studies have demonstrated that abrupt transitions to a solid feed diet induced short-term villus atrophy and crypt hyperplasia, which in turn impaired the digestive efficiency and gut integrity of pigs (van Beers-Schreurs et al., 1998; Hu et al., 2013). In the present study, we discovered that the solute carrier family, including many proteins that function as transporters of a large variety of molecules, was down-regulated by the weaning process, such as the mitochondrial carrier (*SLC25A16* and *SLC25A4*), nucleotide-sugar transporter (*SLC35B4*), sodium- and chloride-dependent sodium: neurotransmitter symporters (*SLC6A4*), and Na^+^/H^+^ exchanger (*SLC9A2*). Furthermore, we observed that the mineral absorption pathway was decreased in the weaning process. These results demonstrated that the function for nutrient absorption in the intestine of piglets was impaired in the weaning process.

Weaning stress includes dietary and environmental transitions, and separation from the dam has been demonstrated to disrupt antioxidant defense capacity and cause oxidative stress in piglets (Yin et al., 2014; Ji et al., 2019). In the present study, DEGs related to the oxidation-reduction process and oxidoreductase activity were identified by GO enrichment analysis, which may elucidate the molecular mechanisms of oxidative stress induced by early weaning. *FDXR*, the mitochondrial cytochrome P-450 NADPH reductase, was increased by the weaning process. A previous study has demonstrated that *FDXR* is a target gene of the tumor protein 53 (p53) family and that increased levels of reactive oxygen species (ROS) can activate p53, which induces the expression of *FDXR* (Liu and Chen, 2002). Tumor protein 53 (p53), which regulates the generation of reactive oxygen species, was enhanced in the jejuna of piglets after weaning (Yin et al., 2014). To protect cells from the damage caused by ROS, organisms have evolved several defense mechanisms to rapidly and efficiently remove ROS from the intracellular environment. The antioxidant enzyme system, including SOD, CAT and GPX, is the first line of defense against ROS in organisms. SOD performs efficient dismutation of O_2_^–^ to H_2_O_2_, which can be reduced to water by GPX and CAT. In the present study, *SOD3* and *GPX2* were upregulated in weaned piglets, indicating that the antioxidant enzyme system was activated to prevent oxidative stress induced by the weaning process. However, we found that *CAT* expression in the gut of weaned piglets was down-regulated, demonstrating that the activated antioxidant enzyme system was damaged by the weaning process. Decreased *CAT* expression may result in the accumulation of H_2_O_2_. Although H_2_O_2_ is not a radical itself, this molecule is reactive and is rapidly converted to the highly reactive ^•^OH via the Fenton reaction unless efficiently removed. The ^•^OH radical is widely accepted as being the most damaging ROS produced by cells (Rodriguez et al., 2004). In addition to *GPX2* and *SOD3*, *CAT* was found to interact with *ACADSB*, *HSD17B4*, *IYD* and *LDHA* (Figure 5), which indicated that *CAT* may further influence other genes related to the oxidation-reduction process and oxidoreductase activity. Among these genes, *LDHA* (lactate dehydrogenase A), which converts pyruvate to lactate coupled with the recycling of NAD^+^, was down-regulated by the weaning process. A previous study demonstrated that reduction of *LDHA* would favor the entry of pyruvate into mitochondria for oxidative phosphorylation, thereby enhancing oxygen consumption and inducing oxidative stress (Le et al., 2010).

The intestinal microbiota of young animals has a profound influence on the establishment of the gut barrier, intestinal nutritional metabolism and immune system maturation (Merrifield et al., 2016). Aberrations in the composition and function of the gut microbiota, termed microbiota dysbiosis, are important factors that affect gut gene expression. In the present study, 16S rRNA sequencing was used to measure the microbiota composition. In addition, the functions of the intestinal microbiota of piglets were examined by predicting the metagenomes using PICRUSt analysis. As predicted, the gut microbiota composition and the function were significantly shaped by early weaning. Firmicutes and Bacteroidetes are the two predominant phyla in the gut microbiota of pig and human (Li et al., 2018). However, the intestinal microbiota of human infants and young piglets is not stable like that of adult humans and pigs. In the present study, we observed that the abundance of Firmicutes and the Firmicutes/Bacteroidetes ratio were higher in weaned piglets than that in sucking piglets. An increasing number of studies have demonstrated that the Firmicutes/Bacteroidetes ratio is related to obesity and that Firmicutes species have an increased capacity to harvest energy from the diet (Ley et al., 2006; Turnbaugh et al., 2006). In addition, we observed the genes *FABP6* and *NR1H4* (also called *FXR*) related to bile acids were increased in the gut of weaned piglets. Bile acids are synthesized in the liver and secreted into the gut in bile after ingestion of food and have emerged as important biological molecules that support the solubilization of various lipids and lipid-soluble compounds in the gut (Lin et al., 2019). *NR1H4* is an important bile-acid-activated and transcriptional regulator gene and plays an important role in bile acid homeostasis. In addition, *FABP6* has been shown to be needed for the efficient transport of bile acids from the apical side to the basolateral side of enterocytes in the distal intestine (Zwicker and Agellon, 2013). In the weaning transition of pigs, the dietary was changed from highly digestible milk to a less digestible solid feed. Thus, the increased Firmicutes and bile acids metabolism may be an adaptive mechanism in weaned piglets for digestion solid feed. But the predictive metagenome showed that pathways related to PPAR signaling and alpha-linolenic acid metabolism were decreased in the gut microbiota of weaned piglets, which may contribute to the decreased gene expression of *CPT2* and *FABP1* in fatty acid metabolism and PPAR signaling in the intestine and further demonstrated that fatty acid transport and oxidation may be inhibited by the early weaning stress.

In addition, we observed that the pathway related to the citrate cycle (TCA cycle) was decreased in weaned piglets by predictive metagenome analysis. Similarly, the gene expression of *MPC2* was down-regulated in the intestine of weaned piglets. *MPC2* is responsible for the transport of pyruvate in mitochondria. Pyruvic acid plays important roles in the TCA cycle and can be made from glucose via glycolysis and converted back to carbohydrates (such as glucose) via gluconeogenesis or to fatty acids via a reaction with acetyl-CoA. Thus, we speculate that the TCA cycle may be inhibited by the weaning process. Interestingly, glutamine synthetase (*GLUL*), which catalyzes the synthesis of glutamine from glutamate and ammonia in an ATP-dependent reaction and participates in pathways of nitrogen metabolism and amino acid metabolism (Table 1), was decreased in the intestine of weaned piglets. The predictive metagenome analysis also showed that the pathway of D-Glutamine and D-glutamate metabolism was decreased in the gut microbiota of weaned piglets. Thus, the decreased *GLUL* expression in the intestine induced by weaning may be due to the changes in gut microbiota composition and their metabolic functions. Previous studies have demonstrated that glutamine plays a critical role in maintaining multiple important functions, such as nutrient metabolism, immune response, and intestinal integrity, as well as the synthesis of other bioactive compounds (Li et al., 2018). Therefore, glutamine supplementation in weaning piglets may be an effective way to improve growth and maintain gut health (Wang et al., 2008; Ji et al., 2019).

Bacteroidetes consists of two main genera, namely, *Bacteroides* and *Prevotella*, and these two genera usually compete for the same niche in the gut, which results in subjects with high levels of *Prevotella* usually having low levels of *Bacteroides* (Kovatcheva-Datchary et al., 2015). *Bacteroides* species have been demonstrated to consume milk oligosaccharides via mucus utilization pathways (Marcobal et al., 2011; Marcobal and Sonnenburg, 2012). *Prevotella* is linked to the fermentation of plant-derived non-starch polysaccharides to SCFAs and produces enzymes, such as β-glucanase, mannase, and xylanase, that can degrade polysaccharides in the plant cell wall (Flint and Bayer, 2008; Guevarra et al., 2018). In addition, high dietary fiber intake has been demonstrated to induce improvement in the abundance of *Prevotella* and the *Prevotella*/*Bacteroides* ratio (Kovatcheva-Datchary et al., 2015). Thus, dietary changes from milk to solid feed with dietary fiber would contribute to the decreased *Bacteroides* abundance and increased *Prevotella* abundance and *Prevotella*/*Bacteroides* ratio, which is consistent with previous studies (Frese et al., 2015; Chen et al., 2017).

SCFAs, which are the end products of dietary carbohydrates, specifically resistant starches, and dietary fiber fermentation by the intestinal microbiota in colon, primarily consist of acetate, propionate, and butyrate (Schwiertz et al., 2010). SCFAs production depends on the substrates flowing into the large intestine and the population and composition of the intestinal microbiota (Haenen et al., 2013). In humans and pigs, weaning transition affects not only the structure and function of the intestine but also the components of the luminal environment, such as the intestinal microbiota and its metabolites (Nakatani et al., 2018). In the present study, the levels of butyrate and acetate were higher in weaned piglets than that in sucking piglets. One reason for this effect is that the abundances of SCFAs-producing bacteria, including *Faecalibacterium*, *Blautia*, *Oscillibacter*, *Roseburia*, and *Prevotella* species, were increased by the weaning process. In humans (Li et al., 2014) and pigs (Salcedo et al., 2016), maternal milk contains large amounts of oligosaccharides, and disaccharides and thus, SCFAs production by hindgut fermentation starts a few days after parturition (Nakatani et al., 2018). Therefore, the fermentative substrates changed with the transition from maternal milk to solid food, which provided increased levels of fermentation substrates and contributed to the increase in SCFAs levels (van Beers-Schreurs et al., 1998). In the present study, we observed that the gene expression of peptide tyrosine-tyrosine (*PYY*) was increased in the weaned piglets (Supplementary Table 4). Interestingly, previous studies have demonstrated that microbial SCFAs, particularly butyrate, cause a dose- and time-dependent increase in *PYY* gene expression in *vivo* (Psichas et al., 2015) and *vitro* (Larraufie et al., 2018) models. In the present study, the change in *PYY* gene expression may be related to the increased SCFAs levels. *PYY* has been shown to induce satiety by upregulation of anorexigenic signal pathway and downregulation of orexigenic signaling molecule neuropeptide tyrosine in the hypothalamus, which is associated with food intake of pigs (Wang et al., 2019a). Thus, the increased SCFAs production and *PYY* gene expression may be an important reason for the reduced feed intake in the weaning transition. In addition, SCFAs can be rapidly absorbed by the intestine and subsequently utilized by the host as substrates for energy production (Morrison and Preston, 2016). SCFAs also contribute to the maintenance of gut morphology and function. Butyrate has been demonstrated to prevent and inhibit gut carcinogenesis, protect against mucosal oxidative stress and inflammation and strengthen the intestinal defense barrier (den Besten et al., 2013). Thus, the increased levels of SCFAs may serve as important roles in the intestinal function during the weaning process.

On the other hand, the abundance of Proteobacteria, including the genera *Sutterella*, *Helicobacter*, *Escherichia*, *Actinobacillus*, was decreased, and *Fusobacteria* nearly disappeared in weaned piglets compared to sucking piglets, which is consistent with previous studies (Chen et al., 2017; Li et al., 2018). Proteobacteria includes a wide variety of pathogenic bacteria and plays important role in immune education in early life. The gradual maturation of gut immune function and the microbiota may contribute to the decrease in Proteobacteria. Recently, studies have shown that Proteobacteria is one of the most dominant phyla in human (Moossavi et al., 2019) and porcine milk (Chen et al., 2018). Although the origin of the bacteria in breast milk is currently not known, bacteria in breast milk may be an important source of the intestinal microbiota of infants and sucking piglets. During the weaning process, a dietary shift from milk to a completely feed-based diet may be an important reason for the decrease in Proteobacteria in weaned piglets.

## Conclusion

The results of the present study demonstrated that weaning transition altered intestinal gene expression involved in nutrient absorption, transportation and metabolic processes in pigs. Weaning transition significantly increased the abundance of Firmicutes and the Firmicutes/Bacteroidetes ratio and decreased the abundances of Proteobacteria and *Fusobacteria* in pig feces. The abundances of *Bacteroides* and *Fusobacterium* decreased markedly, while the abundance of *Prevotella* showed a marked increase, in weaned piglets compared to sucking piglets. The levels of butyrate and acetate in the feces were increased by the weaning process. The intestinal gene expression regulated by weaning transition may be associated with the changes in the gut microbiota composition and SCFAs levels. However, further studies are needed for the interaction between host gene expression and gut microbiota in the weaning process of pigs.

## Data Availability Statement

The datasets generated for this study can be found in the Gene Expression Omnibus (GEO) database: GSM2868043, GSM2868044, GSM2868045, GSM2868049, GSM2868050, and GSM2868051.

## Ethics STatement

The animal study was reviewed and approved by the Ethical and Animal Welfare Committee of Heilongjiang Province, China the Ethical and Animal Welfare Committee of Northeast Agricultural University.

## Author Contributions

AS, BS, and QWM conceived and designed the experimental plan. QWM, ZYL, CC, and SS were involved in the animal experiments, analysis, and data collection. QWM, QQM, and ZL analyzed the data and drafted the original manuscript. All authors read and approved the final manuscript.

## Conflict of Interest

The authors declare that the research was conducted in the absence of any commercial or financial relationships that could be construed as a potential conflict of interest.

## Abbreviations

*ACADSB*: acyl-CoA dehydrogenase short/branched chain
*ACAD11*: acyl-CoA dehydrogenase family member 11
*CAT*: catalase
*CRABP1*: cellular retinoic acid binding protein 1
*FABP1*: fatty acid-binding protein 1
*FABP6*: fatty acid-binding protein 6
*FDXR*: ferredoxin reductase
*GPD1*: glycerol-3-phosphate dehydrogenase 1
*GPD1L*: glycerol-3-phosphate dehydrogenase 1 like
*GPX2*: glutathione peroxidase 2
*HSD11B2*: hydroxysteroid 11-beta dehydrogenase 2
*HSD17B11*: hydroxysteroid 17-beta dehydrogenase 11
*HSD17B12*: hydroxysteroid 17-beta dehydrogenase 12
*IYD*: iodotyrosine deiodinase
*LDHA*: lactate dehydrogenase
*MPC2*: mitochondrial pyruvate carrier 2
*MOCS3*: molybdenum cofactor synthesis 3
*P4HTM*, prolyl 4-hydroxylase: transmembrane
*PYY*: peptide tyrosine-tyrosine
*SOD3*: superoxide dismutase 3
*TET2*: tet methylcytosine dioxygenase 2.
