# Supplementary material for: Weaning Alters Intestinal Gene Expression Involved in Nutrient Metabolism by Shaping Gut Microbiota in Pigs
Source: Front Microbiol. 2020 Apr 17;11:694. doi: 10.3389/fmicb.2020.00694 (PMC7181064; doi:10.3389/fmicb.2020.00694)
Supplement: Supplementary file 1 [file Data_Sheet_1.docx]

Supplementary Material

# Supplementary Table 1. Primers used for qRT-PCR.

| Genes | Sequence (5'to3') | Product Size (bp) |
| --- | --- | --- |
| *CAT* | F: ACGCCTGTGTGAGAACATTG | 124 |
|  | R: GTCCAGAAGAGCCTGAATGC |  |
| *CPT2* | F: GGCTTTGACCGACACTTGTT | 119 |
|  | R: CTGGTGGACAGGATGTTGTG |  |
| *FABP1* | F: GGTCCAAGGTCGTCCAGAATGAG | 159 |
|  | R: TGTCACCGTTGAGTTCAGTCACAG |  |
| *LDHA* | F: TGCTTGTTGCTTCCAATCCAGTGG | 200 |
|  | R: AGGCACACTAGAGTCTCCATGCTC |  |
| *GPX2* | F: CTCGCTCTGAGGCACAACCAC | 160 |
|  | R: GCGGACGTACTTGAGGCTGTTC |  |
| *FDXR* | F: TCGGACAGCCACGGAGAAGC | 152 |
|  | R: TGGTGACTGCCAGGCGGATG |  |
| *SOD3* | F: ACGCTGCTCTGTGCTTACCT | 135 |
|  | R: CTGCCAGATCTCCGTCACTT |  |
| *SLC1A5* | F: TCCTGGTGCTGCCTCTCATCTAC | 146 |
|  | R: CTCCTCCACGCACTTCATCATCAG |  |
| *GAPDH* | F: ATGGTGAAGGTCGGAGTGAA | 155 |
|  | R: CCGTGGGTGGAATCATACTG |  |

**Supplementary Table 2. Summary of sequencing analysis in RNA-seq.**

| Item | Raw Data | Valid Data | Valid Ratio (%) | Q20 (%) | Q30 (%) | GC content (%) |
| --- | --- | --- | --- | --- | --- | --- |
| S-1 | 40418456 | 39763038 | 98.38 | 98.44 | 86.01 | 53.00 |
| S-2 | 41946130 | 41257126 | 98.36 | 98.96 | 88.32 | 53.00 |
| S-3 | 50070676 | 49301698 | 98.46 | 99.23 | 89.92 | 53.50 |
| W-1 | 40736316 | 40224050 | 98.74 | 98.92 | 87.70 | 54.50 |
| W-2 | 57292800 | 56591170 | 98.78 | 99.03 | 88.34 | 55.50 |
| W-3 | 47328436 | 46618492 | 98.50 | 99.03 | 89.07 | 54.50 |

S: Sucking piglets, n=3; Weaned: Weaned piglets, n=3. Q20: the percentage of bases with a Phred value > 20; and Q30: the percentage of bases with a Phred value > 30

**Supplementary Table 3. The data for the sequencing reads that mapped to the reference genome in RNA-seq.**

| Sample | Valid reads | Mapped reads | Unique Mapped reads | Multi Mapped reads | PE Mapped reads | Reads map to sense strand | Reads map to antisense strand |
| --- | --- | --- | --- | --- | --- | --- | --- |
| S-1 | 39763038 | 30826998 (77.53%) | 20486031 (51.52%) | 10340967 (26.01%) | 27497000 (69.15%) | 14378466 (36.16%) | 14483587 (36.42%) |
| S-2 | 41257126 | 32619837 (79.06%) | 22301313 (54.05%) | 10318524 (25.01%) | 29372956 (71.19%) | 15243066 (36.95%) | 15317608 (37.13%) |
| S-3 | 49301698 | 39481643 (80.08%) | 26643827 (54.04%) | 12837816 (26.04%) | 36338478 (73.71%) | 18351520 (37.22%) | 18395691 (37.31%) |
| W-1 | 40224050 | 30628190 (76.14%) | 18096060 (44.99%) | 12532130 (31.16%) | 27005628 (67.14%) | 14280422 (35.50%) | 14398367 (35.80%) |
| W-2 | 56591170 | 43889785 (77.56%) | 26029805 (46.00%) | 17859980 (31.56%) | 38983310 (68.89%) | 20454502 (36.14%) | 20564962 (36.34%) |
| W-3 | 46618492 | 35880287 (76.97%) | 22553568 (48.38%) | 13326719 (28.59%) | 32413472 (69.53%) | 16741557 (35.91%) | 16883575 (36.22%) |

S: Sucking piglets, n=3; W: Weaned piglets, n=3.

**Supplementary Table 4. Differentially expressed genes (DEGs)** **between sucking piglets and weaned piglets.**

As shown in Supplementary Material 2.

**Supplementary Table 5. GO enrichment analysis of differentially expressed genes (DEGs) between sucking piglets and weaned piglets.**

As shown in Supplementary Material 3.

**Supplementary Table 6: Summary of sequencing analysis in 16s rDNA.**

| Item | Raw Data | | Valid Data | | Valid % | Q20 % | Q30 % | GC % |
| --- | --- | --- | --- | --- | --- | --- | --- | --- |
|  | Tag | Base | Tag | Base |  |  |  |  |
| S-1 | 41872 | 19.85M | 41071 | 16.90M | 98.09 | 97.11 | 89.89 | 50.85 |
| S-2 | 50736 | 24.05M | 47217 | 19.28M | 93.06 | 97.19 | 90.01 | 53.02 |
| S-3 | 30498 | 14.46M | 29798 | 12.03M | 97.70 | 97.54 | 91.03 | 50.49 |
| S-4 | 37218 | 17.64M | 36307 | 14.74M | 97.55 | 96.99 | 89.56 | 52.87 |
| S-5 | 42037 | 19.93M | 34013 | 13.77M | 80.91 | 97.07 | 89.86 | 52.09 |
| W-1 | 40434 | 19.17M | 35252 | 14.42M | 87.18 | 97.20 | 90.12 | 52.31 |
| W-2 | 30711 | 14.56M | 29595 | 12.07M | 96.37 | 96.98 | 89.51 | 53.24 |
| W-3 | 33669 | 15.96M | 30949 | 12.54M | 91.92 | 97.06 | 89.67 | 52.76 |
| W-4 | 43816 | 20.77M | 33159 | 13.57M | 75.68 | 96.97 | 89.52 | 52.23 |
| W-5 | 40433 | 19.17M | 34201 | 13.88M | 84.59 | 97.22 | 90.26 | 52.01 |
| W-6 | 50234 | 23.81M | 46294 | 18.83M | 92.16 | 97.07 | 89.78 | 52.68 |

S: Sucking piglets, n=5; W: Weaned piglets, n=6.

**Supplementary Figure 1.** **Category of cellular component in GO enrichment analysis.**


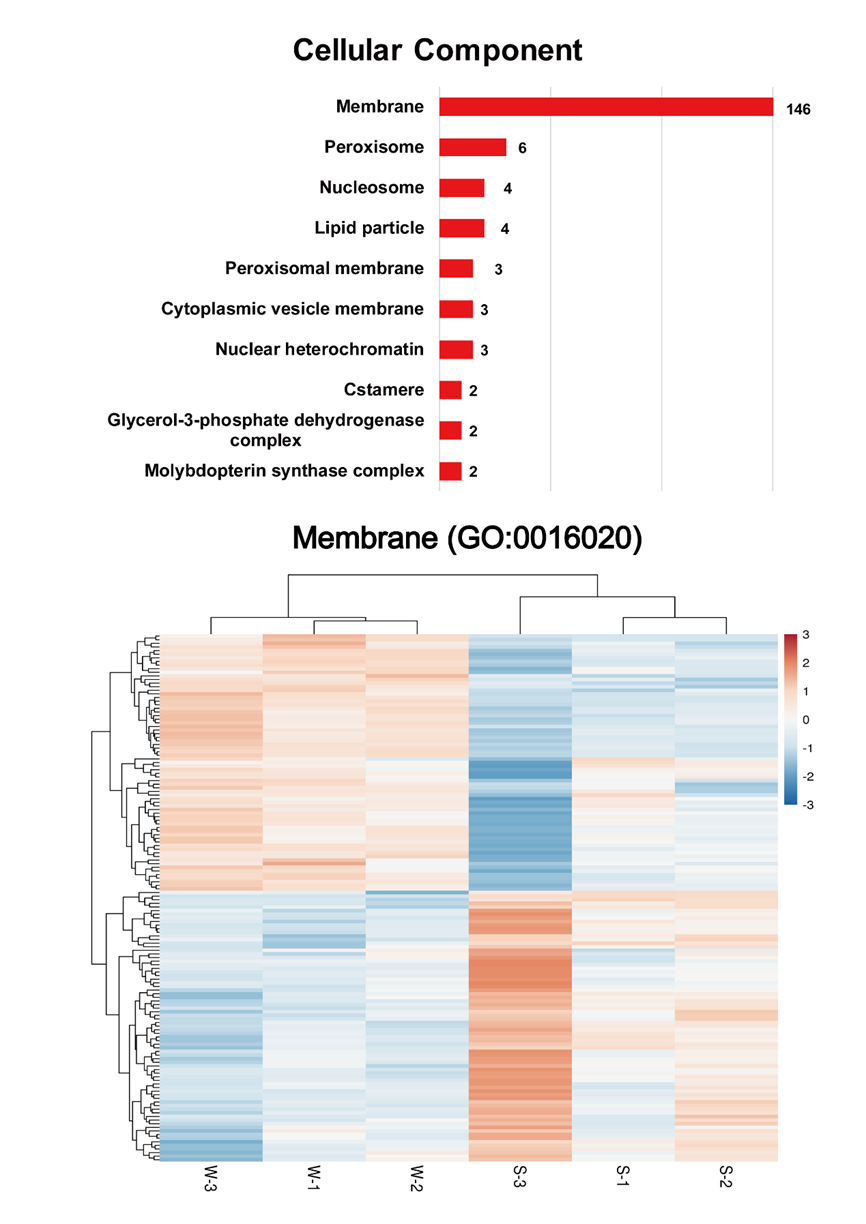


**B**

**A**
